# PeTroShare: Blockchain-Managed, Logistics-Aware, Privacy-Friendly, Comparative, and Efficient Petroleum Transportation

**DOI:** 10.3390/s21217066

**Published:** 2021-10-25

**Authors:** Salwa M. Hassan, Mohamed Azab, Amal O. Hamada

**Affiliations:** 1 Electrical Engineering Department, Faculty of Engineering, Alexandria University, Alexandria 21519, Egypt; salwa.mohamed@mena.vt.edu (S.M.H.); amal.o.hamada@mena.vt.edu (A.O.H.); 2 Department of Computer and Information Sciences, Virginia Military Institute, Lexington, VA 24450, USA

**Keywords:** crude oil, transportation, IoT, block chain, logistics, ride sharing

## Abstract

Crude oil is one of the critically needed resources. It is the main pillar supporting almost everything we rely on in daily life. Unfortunately, due to many factors, crude oil costs too much. Transportation is one of the critical factors that affect such costs. Due to many environmental risks attached to the transportation process, many countries added very high tariffs to cover any hazards during the transportation, loading, and unloading process. Logistics concerns and political conflicts are the other key factors that can massively impact the transportation cost. This paper presents an Industry 4.0-compliant PeTroShare (PTS), a blockchain-powered trustworthy, logistics-friendly, and cost-efficient crude oil trading platform. PTS is a novel ride-sharing platform that enables an anonymous exchange of crude oil between oil producers and customers, focusing mainly on the product quality, not the source of origin. In our scenario, floating crude oil tankers will hold the cargo to an intermediate position in the open ocean. PTS will match the product availability based on the location and the needed quality of the customer requests. Consequently, the time and distance travelled are minimized. Our simulation results show that enabling the anonymous sharing of crude oil products can significantly enhance system efficiency and cost-effectiveness.

## 1. Introduction

Crude oil is the world’s most actively traded commodity. Generally, it is an essential product in the industry global market. Crude oil is called “black gold”. It is the leader of the industrial revolution and modern technological development. Additionally, it allowed a great development in the means of transportation that provided luxury in human life around the world. Accordingly, the importance of crude oil motivates countries to compete to obtain it [1].

Nevertheless, crude oil is a high-priced commodity due to the transportation process cost. The crude oil transportation process is vital for the petroleum industry [2]. It is one of the critical factors that controls the overall cost of crude oil. There are several methods of crude oil transportation such as rail cars, trucks, tanker vessels, or through pipelines. Tanker vessels are considered the most efficient way to transport crude oil [3]. They accommodate a higher load and transport it with high speed and lower cost.

Climatic and marine fluctuations may affect the balance of tankers which may lead to crude oil leakage. This may cause massive contamination, which threatens marine life [4]. Therefore, petroleum companies increase added tariffs to cover the insurance cost for transportation against transportation hazards. Furthermore, international politics and logistics concerns have an effect on transportation and its cost [4].

Even though the overall cost of crude oil as a product depends on the cost of transportation, the optimization of the transportation process is considered a critical problem and is extremely important.

This paper presents an Industry v4.0-compliant PeTroShare (PTS) platform. PTS is a blockchain-powered trustworthy, logistics-friendly, and cost-efficient crude oil trading platform. PTS can promote the global market by opening a new market for crude oil exchange. It enables crude oil to be exchanged as a public commodity away from ports through the open ocean. PTS is a novel transportation sharing mechanism that facilitates anonymous transactions between crude oil vendors and stakeholders. It enables multiple clients to share the same tanker. Consequently, it minimizes the distance traveled from the vendor to stakeholders’ locations. As a result, the transportation process becomes faster and less costly.

Moreover, human intervention in the monitoring system may cause errors. besides, it may lead to a single point of control. As a result, most disasters are currently more serious due to the existence of a single point of control [5]. PTS monitors the movement of crude oil tankers across the oceans to detect, resolve, and report any disaster that may occur during the transportation process.

The substantial objectives of the presented system (PTS) is to convert the international petroleum trade into a public commodity by automating the role of brokers in the transportation process.The proposed system relies on three pillars, IoT networks, ride sharing, and blockchain technology.

**Figure 1:**
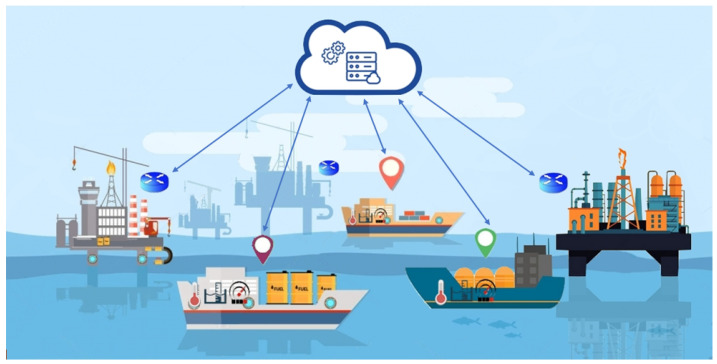
PTS system overview.

The first pillar integrates a cooperative IoT network with the crude oil transportation system. The IoT network is composed of connected tankers with IoT sensors scattered across the transportation tankers for quality control and location tracking, as shown in Figure 1. The current tankers are equipped with sensors and actuators for different purposes such as temperature monitoring and crude oil level detection. Additionally, there is a wireless communication system for navigation. PTS uses the smart systems that are already in modern ships, which are built to monitor the behavior of its components. Modern ships were built to update operators on shore on such aspects [6]. So, the infrastructure needed is already there.

The presented approach relies on IoT network feedback which guides a multi-objective optimization process to minimize transportation costs and maximize the quality of service. In addition, we assume that a navigation system is embedded in oil tankers that uses GPS signals to send the tanker’s current location and its direction to the data warehouse running on the cloud. This monitoring system assists in selecting the optimal tanker for the customer with efficient cost. In addition, the problems during transportation such as changing the oil temperature or density lead to change the oil characteristics and affect its volume or quality. Oil leakage is the most critical problem that might happen. PTS mitigates the consequences of this problem by sending alarm signals to the nearest convenient tankers to help in handling the problem.

The second pillar is ride sharing offered as a service in the crude oil transportation process through the open ocean. PTS uses a ride-sharing service to exchange multiple client petroleum products in the open ocean. Ride-sharing decisions depend on the IoT devices’ feedback about the tanker location and trajectory and crude oil quantity and quality. Ride sharing facilitates crude oil transportation in intermediate positions between the source and destination to decrease the distance between the vendor and customer. As a result, the transportation process becomes faster and less costly.

The third pillar of PTS relies on a decentralized approach to achieve the aforementioned objective by integrating the blockchain technology with the PTS architecture. Therefore, there will be no central establishment controlling petroleum trade worldwide. PTS exploits the blockchain to preserve a trustworthy anonymous transportation process. Blockchain has the full potential of establishing a trusted and decentralized transportation system. PTS proposes a smart logistics-friendly framework to decrease human intervention and preserve the reliability of the transportation process. This framework will have all the information about the transportation process and total service cost.

The main contributions of the paper are summarized as follows:

1. Presenting a novel framework to facilitate safe, logistics-aware, privacy-friendly, cooperative, and efficient crude oil cargo sharing.
2. Enabling blockchain-based monitoring and management for petroleum transportation to facilitate a safe and cost-efficient operation.

The rest of this paper is organized as follows: Section 2 shows a detailed literature review about the related work of the crude oil transportation process. Section 3 introduces the proposed system. Section 4 introduces the searching algorithm and optimization function, which are given in detail. Then, results are provided in Section 6. Finally, the conclusions and ideas for future work are presented in Section 7.

## 2. Related Work

Integrating IoT devices in industry has facilitated monitoring the industrial aspects of the industrial process and collecting massive amounts of data for further processing [7]. Consequently, combining cooperative IoT networks in the transportation process enhances the quality of service and decreases transportation costs.

In [8], authors proposed an approach to support IoT-based energy management in smart factories. IoT devices are used to collect energy consumption data and then this data have been analyzed and provided to decision makers. They used a pilot study in order to test the proposed approach. Eventually, integrating energy data in production management reduced the wastes and enabled energy-aware decision making at the production management level.

In [9], the authors proposed an IoT application for logistics and supply chain management services, such as vehicle fleet tracking, goods monitoring, and control, location-based services. They used IoT-enabling technologies and grouped them according to functional blocks. These functional blocks are used to facilitate various utilities to the system such as sensing identification, actuation, communication, and management. As a result, this application improved service offerings and enhanced route travel patterns and transport conditions.

Researchers in [10] reviewed and analyzed various modes and applications for IoT in freight transportation, warehousing, and delivery. Smart freight transportation based on IoT as a framework facilitated the transportation of goods. IoT technology made freight transportation more efficient, convenient, and visualized.

Additionally, IoT is used to solve crude oil transportation problems in the Arctic zone [11]. NSR and SCR models (profit decision models) are analyzed to compare transportation costs while using different types of ships. They also have to consider the high variability of the crude oil barrel price and provide a safe means of transportation to avoid the risk of an oil spill due to ice. Although their results illustrated that the use of the NSR is a solution to shipping a larger cargo compared to the SCR, they did not avoid the risks represented by ice drifting and the necessity to use ice-class vessels.

In addition, enabling the ride-sharing concept in transportation allows faster and less costly processes. The authors of [12] proposed a privacy-preserving ride-matching scheme. This scheme is used for selecting feasible ride-share partners in ride-sharing services. Their system is proposed to address the conflict between privacy leakage and partner selection. They can significantly reduce the total transport time cost for both drivers and riders and cut the threats to their privacy leakage. Additionally, the ride-share matching and routing problem in a nonprofit P2P ride-sharing system consists of a matching agency, drivers, and riders. The matching agency is a government or a not-for-profit organization, and its objective is to maximize the societal benefits of ride sharing [13].

The authors of [14] introduced PrivatePool, a model for privacy-preserving ride-sharing application. They investigated ride-sharing patterns that have demonstrated the benefits of a two-fold approach: (i) ride-matching based on the proximity of start and endpoints of the rides and (ii) ride-matching based on the overlap of the ride trajectories. To find the proper ride-share match, the ride requests are further filtered based on the positive transport time saving calculated from the map distance, ensuring the ride-share match can save time and fuel.

Researchers in [15] proposed an application for smart parking. They integrate a ride-sharing service in their application in order to redistribute the capacity of different parking garages. In addition to this, they used IoT technology to indicate vacant spots and send their locations to drivers. Integrating the ride-sharing concept in their system facilitated the transportation process and decreased total cost.

The authors of [16] presented ride sharing as a fast taxi searching algorithm using a spatio-temporal index to quickly retrieve candidate taxis. They proposed two techniques of searching algorithms (single-side taxi searching and dual-side taxi searching). Furthermore, they compared the performance of one non-ride-sharing method and four versions of the proposed ride-sharing method. Based on these methods, the experimental results elucidated the effectiveness and efficiency of their system in serving dynamic queries. Their ride-sharing service saved the total travel distance of taxis when delivering passengers. Compared to the single-side taxi searching algorithm, the dual-side taxi searching algorithm decreased the computation cost.

An intensive investigation of a ride-sharing system and a public transit system, which can significantly enhance the mobility and increase the use of public transport, has been studied in [17]. They presented a solution approach to optimally create single or multi-modal ride-share matches. They considered the use of ride sharing as a feeder system for scheduled transit. They extended their work by also allowing transfers to a transit service with a fixed schedule.

Blockchain is a foundational technology that can preserve a trustworthy anonymous transportation process. The authors of [18] presented a reputation system for intelligent transportation systems. Their system computed the optimal routes and counted the user’s reputation. Taking into consideration that the information found in the blockchain is accessible to all users in that any user can query the system regarding the optimal route between two locations, they defined the blockchain as open “permissionless”. As a result, they finally provided users with an optimal travel route based on reliable data, as well as maintained confidentiality. Additionally, integrating IoT in the transportation process leads to said process being vulnerable to security attacks. The authors of [19] proposed a layered framework BCTLF that integrates IoT and blockchain in transportation and logistics to make it efficient and resilient against several security attacks. They presented two real-life IoT and blockchain-based case studies to highlight the contribution of IoT and blockchain in logistics and transportation.

In this paper, we present the Industry 4.0-compliant PeTroShare (PTS), a blockchain-powered, trustworthy, logistics-friendly, cost-efficient crude oil trading platform, as shown in Table 1. We allow tankers to perform product exchange through the open ocean with more convenient routes. Furthermore, PTS aims to minimize the cost of the crude oil transportation process and make crude oil a public commodity.

## 3. The Proposed System

As crude oil is one of the crucial traded commodities, the international trading of petroleum currently comprises many stages. In addition, some third-party agents play vital roles to enable a successful transportation process. These agents are called brokers. Brokers play an essential role in presenting various offers to customers from different vendors. The latter customer accepts the offers that fit his requirements.

In addition, international politics and logistics concerns prevent some countries from trading and even transiting their ships through regional borders. Consequently, some countries act as brokers to transport crude oil between the conflicting nations. In some cases, there is no direct marine path between countries to exchange petroleum. Accordingly, they rent containers from broker agents to temporarily store the crude oil and change the method of transportation, most likely through pipelines.

The existence of brokers increases the cost of crude oil transportation. As a result, the PeTroShare (PTS) system’s goal is to present crude oil as a public commodity by automating the role of brokers in the transportation process. PTS is a novel Industry v4.0 solution that exploits IoT networks integrated with the crude oil transportation process. PTS is a fully dynamic transportation system. The presence of IoT sensors, which are embedded in tankers, provides PTS with real-time feedback about a tanker’s location and crude oil status.

The petroleum transportation industry is always risky and affected by different factors [24,25]. There are different scenarios that could happen. Currently, PTS can exploit the real-time IoT network’s feedback to handle leakage problems that might happen during the transportation process by sending alarm signals to the nearest tankers or coast guards to help in handling the problem. Additionally, PTS can mitigate the consequences of the marine piracy problem by enabling the real-time monitoring of the product transportation process. PTS can send immediate alerts if any suspicious actions are detected within the vicinity of the tankers.

**Figure 2:**
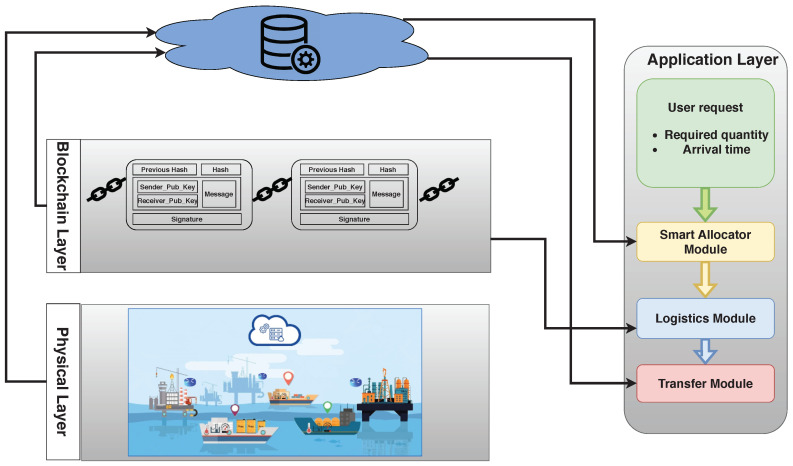
PTS system architecture and modules.

Additionally, the PTS system is a location-independent system which enables the customer to reach his request regardless of the customer’s current location. PTS consists of three modules (smart resource allocator module, logistics module, transfer module), as shown in Figure 2. The smart resource allocator module aggregates customers’ requests to meet customers’ needs. Customer requests include the required crude oil quantity and determined delivery time. Consequently, a smart resource allocator searches for the nearest tanker that has the required crude oil quantity. Then, the logistics module selects appropriate crude oil rigs according to international politics. Additionally, it is responsible for the coordination between the vendor and customer and follows up an end-to-end transportation process. After that, the transfer module facilitates the ride-sharing process within an acceptable time frame. Moreover, it enables the anonymous sharing of petroleum products.

Currently, end-to-end transportation means that the tanker will deliver the requested crude oil from the vendor to the customer itself. There is no possibility of exchanging the products at a crosscut point in the open ocean or even in any other regional water near to the customer. Vendors directly deliver the crude oil to the customers.

### 3.1. Smart Resource Allocator Module

**Figure 3:**
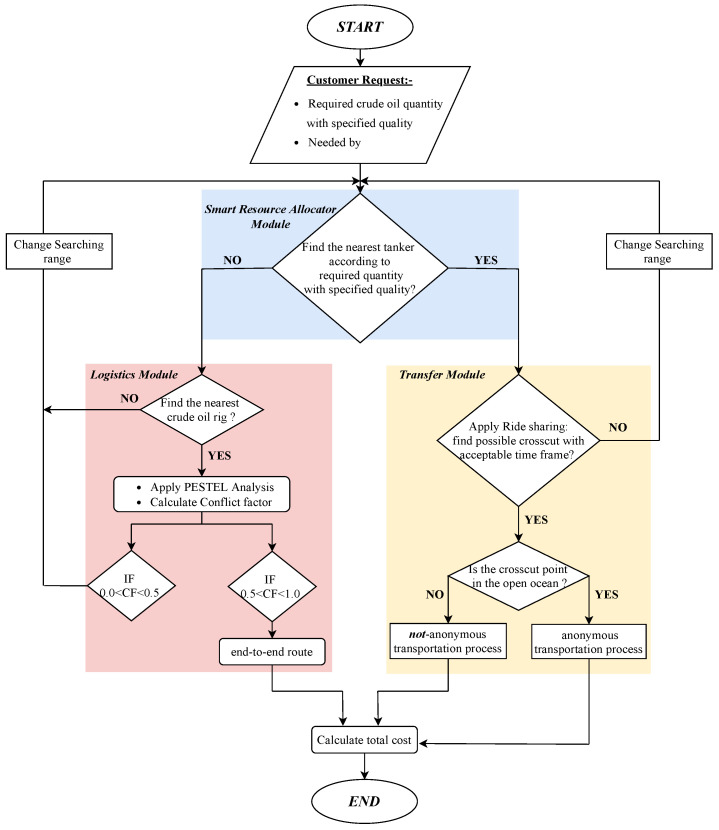
PTS system logic and flowchart.

PTS’s main goal is to perform product exchanges through the open ocean. Therefore, the smart allocator searching process relies on selecting an appropriate tanker that has the required crude oil quantity. First, the smart allocator process starts with receiving customer requests. Then, it searches for appropriate tankers within the starting range, as illustrated in Figure 3. The searching process for the nearest tankers depends on the recorded locations of different tankers. Finally, the smart allocator shows a suitable set of tankers. This set will be filtered again according to the constraints discussed below to implement customer requests. In contrast, if there is not a tanker with the required quantity, PTS searches for a crude oil rig in the same starting range. Otherwise, if the smart allocator does not find the required crude oil in the starting range, PTS will change the searching range and repeat the previous steps.

### 3.2. Logistics Module

Logistics management is a part of supply chain management. It is responsible for the coordination between the vendor and customer and keeps an eye on the transportation process. It typically includes transportation management, fleet management, materials handling, order fulfillment, and supply/demand planning.

In the PTS system, the logistics module is responsible for coordinating a crude oil rig and customer and the follow-up ocean freight process. This module is responsible for handling the end-to-end route according to the political issues between the selected crude oil rig and the customer. Therefore, the transportation process depends on the data collected by the IoT sensors which will be stored in a data warehouse on the cloud. These data contain crude oil rigs’ locations.

First, the logistics module searches for the nearest crude oil rig in the starting range. If there is not a crude oil rig in the starting range, PTS will change the searching range, as illustrated in Figure 3. Then, the logistics module selects an appropriate crude oil rig using PESTEL analysis [26]. PESTEL analysis is a technical framework of macro-environmental factors (political, economic, social, technological, environmental, and legal). It is used in the environmental scanning component of strategic management whereby an organization can assess major external factors that influence its operation in order to become more competitive in the market.

Dependencies and relations between PESTEL factors are determined by the following steps:

1. Identifying PESTEL factors (political, economic, social, technological, environmental and legal);
2. Mapping the interdependencies between PESTEL factors by DEMATEL (decision making trial and evaluation laboratory);
3. Calculating the macro-environment level.

In the PTS system, PESTEL analysis is a simple and effective tool used in situation analysis to select an appropriate vendor, a crude oil rig. The data on the aforementioned factors can be found on websites such as trading economics [27], the global economy [28], data OECD [29], and data world bank [30]. The PTS system uses these data to calculate the macro-environment level. The macro-environment level represents relationships between countries and their logistics concerns. So, the conflict factor is calculated to represent the level of disagreement between customers and vendors who participated in the international petroleum trade using our presented system PTS. The conflict factor is a number used to make a decision to deal with a crude oil rig or not [26]. Depending on the value calculated for the macro-environment level, the decisions are made. According to [26], the macro-environment level is between 0.0 and 1.0. Researchers divide this range into several thresholds based on their system designs. For simplification, we divided the range between 0 and 1 into one threshold. We assumed that the threshold value of the conflict factor was 0.5. Therefore, if the conflict factor is bigger than 0.5, PTS will handle an end-to-end route. Otherwise, PTS changes the searching range and repeats the previous steps.

### 3.3. Transfer Module

The transfer module orchestrates the system interaction process between the selected tankers and the customer’s vessel. This module uses a ride-sharing service to transfer petroleum products from the selected tanker to its destination. Furthermore, blockchain is used to handle the anonymous transportation process.

First, the smart allocator sends a set of tankers to the transfer module. Moreover, the PTS system determines the trajectory of the suggested tankers and the customer’s destination according to the GPS signals. Second, the transfer module estimates the nearest crosscut point in an acceptable time frame to apply the ride-sharing process, as shown in Figure 3. If the transfer module cannot find possible crosscuts within an acceptable time frame, the PTS changes the searching range and repeat the previous steps. After that, the transfer module determines whether the crosscut point through the open ocean or in the regional water. If the crosscut point is in the open ocean, the exchange process can be handled through the open ocean. Consequently, PTS controls an anonymous transportation process. Otherwise, the crosscut point will be in regional water. As a result, the transportation process will not be anonymous. Finally, the meeting points will be sent to the customer’s vessel.

PTS calculates the total service cost based on end-to-end/shared service. It is expected that the transportation cost for an end-to-end route is more expensive than a shared route.

According to the aforementioned constraints, the system searches for suitable tankers that meet the customer’s requirements, ensuring the availability of the ride-share process for minimal cost. Finally, the PTS system provides the selected tankers with information such as their location and the route service details.

## 4. Searching Algorithm

The purpose is to build a model that provides optimal decisions according to the system constraints. Searching algorithm description is shown in Algorithm 1. Suppose there is a customer request ($R_{c}$). This request contains the required quantity ($Q_{r}$) that is needed by and delivery time (*T*). The requests from different customers are aggregated by the PTS system. PTS has a set of requests $S_{r}=\left\{R_{c1},R_{c2},R_{c3},\cdots \right\}$. In addition, PTS records customers destinations ($L_{c}$) and requests submission time ($T_{sub}$). Moreover, the data warehouse includes set of crude oil rigs $S_{o}=\left\{O_{r1},O_{r2},O_{r3},\cdots \right\}$, their location ($L_{o}$) and set of crude oil tankers through open ocean $S_{T}=\left\{C_{t1},C_{t2},C_{t3},\cdots \right\}$, tanker trajectory and available quantity of crude oil ($Q_{a}$). Table 2 indicates the used list of notations in the searching algorithm.
**Algorithm 1:** Searching Algorithm
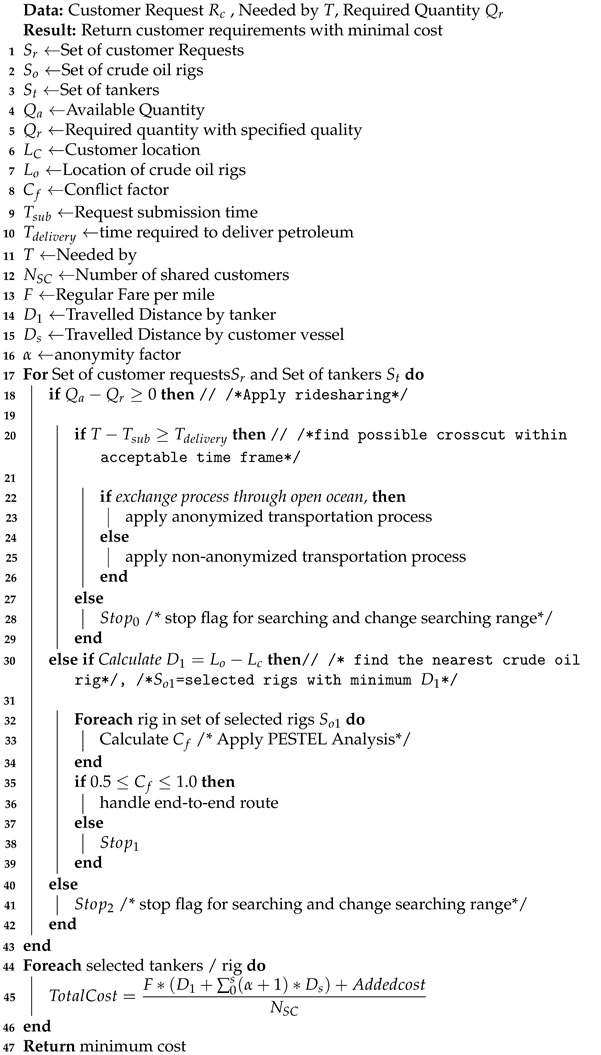


Our searching algorithm procedures start with smart resource allocator which will search for the nearest tanker that has the required quantity. Any other arbitrary tanker is selected by the searching algorithm if and only if Equation (1) holds, where $Q_{a}$ represents the available quantity of crude oil in any tanker. Equation (1) indicates that the available quantity of crude oil in the selected tanker should exceed the crude oil quantity required by the customer.

(1) $$Q_{a}-Q_{r}\geq 0$$

To find all the nearest tankers that hold Equation (1), the searching algorithm simply tests all tankers in the nearest range of customer locations. Then, the set of tankers that holds Equation (1) are candidate tankers.

After that, the transfer module filters a selected set of tankers by the smart resource allocator module. Applying the ride-sharing process depends on an acceptable time frame which is represented by Equation (2) where *T* is the arrival time that the customer would not accept the product if crossed, $T_{sub}$ is the time when customers submit his request and $T_{delivery}$ is the time required to deliver petroleum products to customer destination.

The delivery time $T_{delivery}$ depends on acceptable waiting time for loading/offloading process of petroleum products $T_{wait}$, the tanker trip time required to reach the customer’s vessel $T_{travel}$ and the number of shared customers $N_{SC}$, as represented in Equation (3).

The tanker trip time required to reach the customer’s vessel $T_{travel}$ depends on the time when tanker needs to meet customer at crosscut point and the time when customer need to reach tanker at the crosscut point, as shown in Equation (4).

(2) $$T-T_{sub}\geq T_{delivery}$$

where:

(3) $$T_{delivery}=T_{travel}+T_{wait}*\left(N_{SC}-1\right)$$

(4) $$T_{Travel}=\frac{D_{1}}{Speed\;Of\;Tanker}+\frac{D_{s}}{Speed\;Of\;Customer\;Vessel}$$

Otherwise, the logistics module searches for the nearest crude oil rig. The logistics module selects the nearest rig according to the least distance between rig and customer location Equation (5).

(5) $$D_{1}=L_{o}-L_{c}$$

GPS signals determine locations for customers and vendors. PTS chooses the best route according to GPS feedback and calculates the distance between source and destination locations based on it. In this regard, the distance is calculated straightforward for simplification. So, weather forecasting was not part of the presented constraints.

The logistics module applies PESTEL analysis on selected rigs using updated feedback in the data warehouse to calculate the conflict factor (Cf).

Determining the conflict factor can be achieved in six steps:

1. Analyze PESTEL factors where *P* represents politics issues, *E* represents economy values, *S* represents social issues, *T* represents technological numbers, *L* represents legal issues, and *E* represents environment value to form a (A) matrix.
2. Identify potential dependencies among PESTEL factors by calculating the average matrix A = [$a_{i_{j}}$] is as follows:
  (6) $$a_{i_{j}}=\frac{1}{n}\sum_{1}^{n}P_{i_{j}}^{K},n=1,2,3,...$$
  where $P_{i_{j}}$: represents the degree to which the respondent thinks factor *i* affects factor *j*, *n*: number of crude oil rigs selected by PTS, *K*: the number of Rigs that responds PTS.
3. Normalize the initial direct-relation matrix *D* by *D* = AxS, where
  (7) $$S=\frac{1}{\left(max\sum_{1}^{n}a_{i_{j}}\right)},\;1\leq i\leq n$$
4. Calculate the total relation matrix *T* by
  (8) $$T=D{\left(I-D\right)}^{-1}$$
  where *I* is the identity matrix and *T* matrix provides information on how one factor affects another, it is necessary for a decision-maker to set up a threshold value to filter out some negligible effects. Only the effects greater than the threshold value are chosen by mapping the dataset of (r+c, r−c).
5. Define *r* and *c* as vectors representing the sum of rows and sum of columns of matrix *T*, respectively. Suppose $r_{i}$ is the sum of the ith row in matrix *T*, then $r_{i}$ summarizes both the direct and indirect effects of factor *i* on the other factors. If $c_{j}$ denotes the sum of the jth column in matrix *T*, then $c_{j}$ shows both direct and indirect effects on factor *j* from the other factors. When *j* = *i*, the sum ($r_{i}$ + $c_{j}$) shows the total effects given and received by factor. Thus, ($r_{i}$ + $c_{j}$) indicates the degree of importance that factor *i* plays in the entire system. In contrast, the reciprocal ($r_{i}$−$c_{j}$) describes the net effect that factor *i* contributes to the system. Moreover, if ($r_{i}$−$c_{j}$) is positive, factor is a net cause; if ($r_{i}$−$c_{j}$) is negative, factor *i* is a net receiver or result. The result of that analysis is used as a conflict factor to make decisions in the transportation process.
6. Set a threshold value (0.5) to classify transportation modes as follows:
  (i) 0.5≤Cf≤1.0
    PTS handles an end-to-end route.
  (ii) 0.0≤Cf≤0.5
    PTS will change the searching range and repeats all previous steps.

PTS objective is to minimize the overall cost of transportation process as calculated in Equation (9)

(9) $$TotalCost=\frac{F*(D_{1}+\sum_{0}^{s}\left(\alpha +1\right)*D_{s})+AddedCost}{N_{SC}}$$

where:

- *F* = regular fare per mile;
- $D_{1}$ = travelled distance by tanker;
- $D_{s}$ = travelled distance by customer vessel;
- AddedCost = added cost in case of anonymous transportation process for insurance as PTS performs exchange process in the open ocean;
- $N_{SC}$ = number of shared customers.

α represents a factor for the added cost of anonymity assurances applied within a particular transportation process. It is used to increase the cost of insurance in case of any hazards happening during the transportation process. We assumed that α = 0 in case of anonymous transportation process and α = 1 in case of the non-anonymous transportation process. Therefore, the point of origin and the final destination of your freight are important factors to consider. If the exchange process occurs through the open ocean, the transportation process will be anonymous. Otherwise, PTS will handle the non-anonymous transportation process.

The added cost value is variable and depends on many factors [31,32]. Such factors are the risk level of the selected route and the duration of the trip through the ocean. The added cost is an extra fee added to the transportation cost in case of an anonymous transportation process as PTS performs the exchange process in the open ocean. This fee is for insurance against transportation hazards that could happen in the open ocean.

The US dollar is the standard of international transactions. This means that the daily change in money markets can influence ocean freight rates and must be considered. Most ocean shipments are calculated based on weight [33]. This means that it is most advantageous to completely fill the container carrying your freight. Unless you are shipping less than the container load (LCL), you will be responsible for paying the entire cost charged for the container, even if it is not completely full. Furthermore, added tariffs will be charged totally on one customer. In contrast, PTS divides added tariffs on shared customers. Consequently, the total cost will be minimized.

(10) $$\eta_{PTS}=\frac{|cost_{shared}-cost_{end-to-end}|}{cost_{end-to-end}}$$

Equation (10) represents system efficiency ($\eta_{PTS}$). It indicates the efficiency of ride sharing in the transportation process. It measures how much cost is saved compared to the case where no ride sharing is practiced.

## 5. Blockchain-Based Anonymity Module

The database of the petroleum trade contains extremely important information from vendors and customers/requesters. This information includes oil field locations, the national income of countries, navigation itinerary, etc. Therefore, it is more vulnerable to any malicious attacks in the open ocean.

Blockchain is a fault-tolerant decentralized technology [34]. There is no central point that could be a vulnerable surface for any attacker. Integrating blockchain with the presented system PTS preserves the privacy of all system parties. Our system is able to synchronize the procurement processes between requesters and vendors in order to expand their commercial horizons.

**Figure 4:**
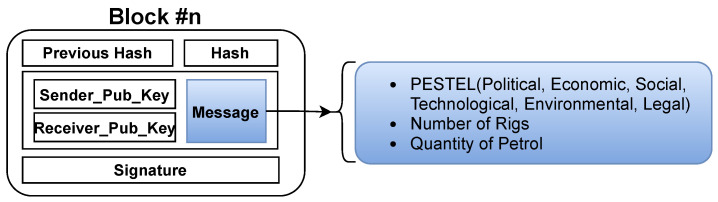
Example for blockchain transaction content.

In this paper, petroleum vendors announce themselves through the blockchain by their real identifications. Each vendor is used as a peer node to build the blockchain network, which is responsible for a piece of information about the availability of crude oil on a certain rig/tanker. Each vendor generates a transaction showing information about its crude oil rigs, traveled tankers and tankers’ trajectory, and the available crude oil quantity, as shown in Figure 4.

In addition, customers generate transactions to show the petroleum products that could be exchanged with others $P_{p}$. PTS integrates vendors and customers as a public petroleum transportation continuum. Although the blockchain is considered as a distributed ledger database, it is not suitable for storing large amounts of data. Consequently, it is linked with the websites [27,28,29,30] to provide the blockchain transaction with vendors and customers’ PESTEL factors without exploiting large storage. Meanwhile, the rigs’ locations and tankers’ availability, scheduling, and navigation are known from the distributed IoT network, as shown in Figure 1. PTS’s main objective is to ensure the confidentiality and reliability of product exchange. To facilitate such goals, PTS relies on the public blockchain scheme to host and process all transactions and operations.

After registration, miners verify the transaction to confirm that vendors and customers are real petroleum authorities. Miners are petroleum authorities with the best conflict factor with other authorities. After the transaction verification, the certificate authority CA issues a pseudonym PID and a hash value *H* to each registered member. A pseudonym is a fake name used to conceal the real identity of the PTS participant. Furthermore, the use of pseudonyms instead of real identities in this network is required to protect the privacy and the identities of participants as well as their locations. These pseudonyms dynamically change by PTS of time to extend their lifetime. The hash is used to facilitate authentication of PTS members. Then, all vendors and customers information is saved on the data warehouse with their pseudonyms, as illustrated in Algorithm 2.

**Figure 5:**
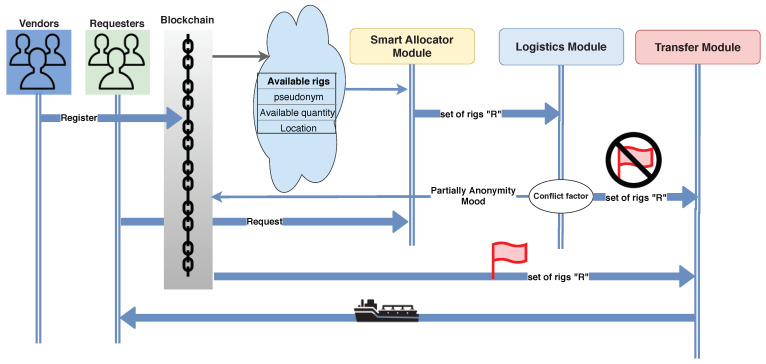
System flow.

Figure 5 shows the system flow starting from vendors’ registration until the required petroleum reaching the requester location. The customer requests a certain amount of petroleum at a certain arrival time ($T_{a}$). The application determines the location of the requester and sends the request to the smart allocator module. The smart allocator module filters the available rigs that meet the customers’ requirements. Subsequently, the filtered set of rigs is submitted to the logistics module. Additionally, the logistics module is informed with the requester information, location, and PESTEL factors. Additionally, the logistics module calculates the conflict factor between the requester and each rig at the filtered set of rigs.
**Algorithm 2:** Blockchain-based anonymity module
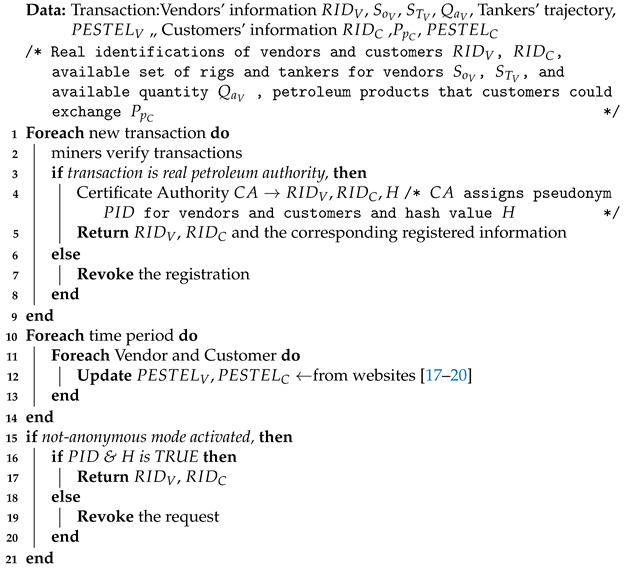


The presented PTS system is considered a conditional anonymity system that depends on the conflict factor value and the location of petroleum products exchange point. In the case of the anonymous mode, vendors and customers deal with each other by their pseudonyms PID. Otherwise, in the non-anonymous mode, the transfer module requests the real identifications RID of crude oil transportation partners, as shown in Algorithm 2. The request is made by the pseudonyms and its block hash to verify that there is no tampering occurred. If the pseudonyms and its block hash are not as saved in the blockchain, the request is revoked.

## 6. Evaluation Results

The simulation results evaluate the effect of enabling anonymous sharing of products to enhance system efficiency and cost-effectiveness. A MATLAB code is set up to evaluate the effectiveness and efficiency of the presented approach to optimize crude oil transportation cost.

MATLAB simulations were built to process hypothetical data formulated to mimic real-world scenarios. Searching algorithm and blockchain-based anonymity module are implemented and applied for the generated data.

The MATLAB code works concurrently with a blockchain emulator, which updates the MATLAB code with vendor and customer information. Such information is the available crude oil quantity and quality, rigs’ location, and tankers’ trajectory. Table 3 shows the parameters used in the system model for the optimal route selection.

**Figure 6:**
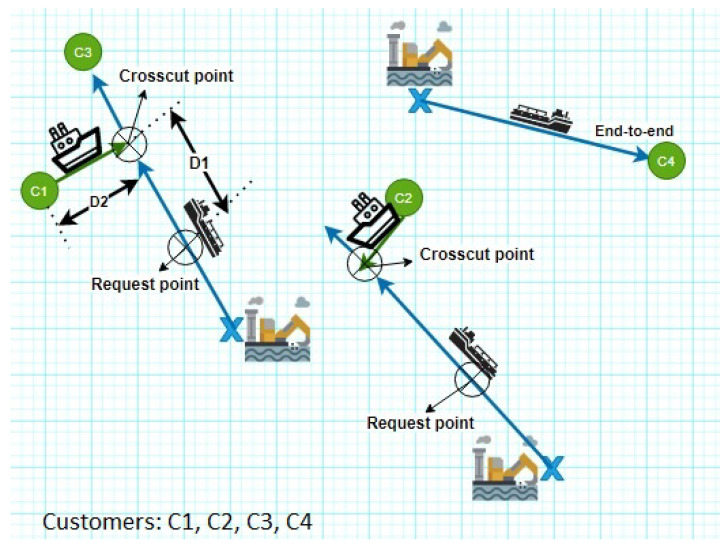
An example of geographical distribution through the ocean.

As an illustration, Figure 6 shows a map of rig locations, tanker trajectories, and the sharing travels according to customers’ requested and the crude oil availability. In this map, the optimal transportation route according to the customer’s requirements is determined using PTS system. PTS locates crosscut points and sends its coordinates to customer’s vessels according to tanker trajectory. Accordingly, the simulation results show the effect of each selected route on the total crude oil transport cost.

The total crude oil transport cost is affected by several factors. These factors are the route type as the PTS could choose either to deliver the crude oil through an end-to-end route or shared route with other customers. It is worth noting that, the number of shared customers has an effect on the total transport cost. In addition, the shared route has two modes: anonymous and non-anonymous routes. However, the oil quantity requested by each customer is included in the searching process and it is a critical factor to find a suitable tanker that could transfer the crude oil, as shown in Equation (1).

**Figure 7:**
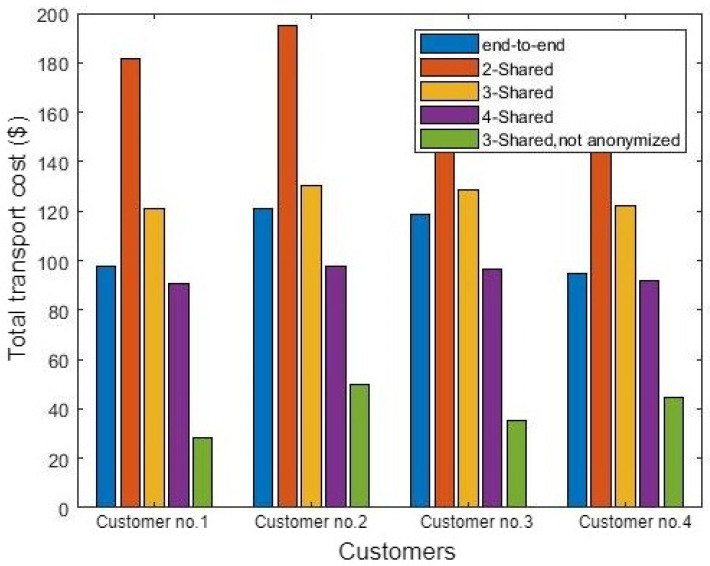
The cost of transportation for the aforementioned cargo.

Figure 7 shows the total transport cost for four customers in different cases such as the end-to-end route, anonymously shared routes with a different number of customers, and a non-anonymous shared route. As illustrated in Figure 7, the non-anonymous shared transportation process is the best selection according to the total transport cost. According to Equation (9), there is no added cost in case of a non-anonymous transportation process, as there is no need for insurance when PTS performs the exchange process in regional water. In addition, the total cost is divided by the number of shared customers sharing the crude oil delivery ride. Therefore, it achieves the minimum cost compared to the end-to-end route or even the anonymous shared route with the same number of customers. In addition, Figure 7 shows that the higher the number of shared customers, the lower the total crude oil delivery cost.

**Figure 8:**
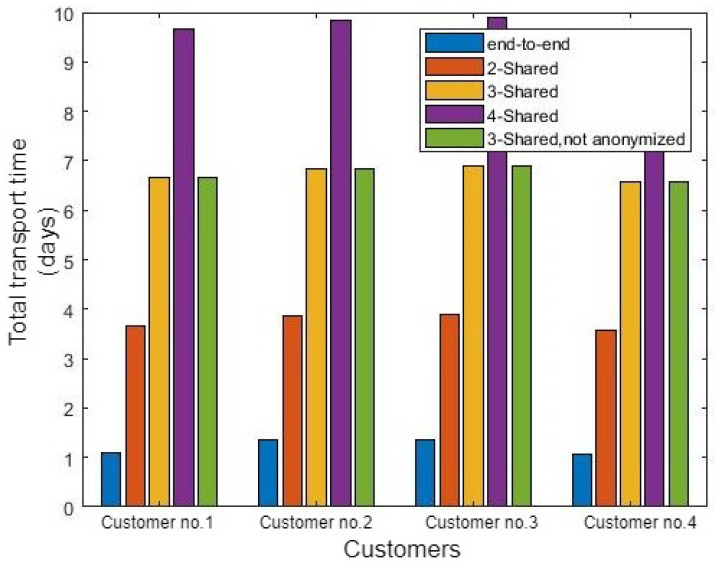
The time of transportation for the aforementioned cargo.

In addition to the factors that affect the total delivery cost of crude oil, some factors affect the time taken to deliver the crude oil to each customer. Otherwise, the best selection according to the cost may not fit all the other preferences such as the time. Figure 8 shows the delivery time variation in various status. As illustrated in Equation (3), the delivery time is affected by transport time and the number of shared customers. Consequently, an increasing number of shared customers led to a delay tanker to reach the destination due to many loading/offloading processes. Therefore, the end-to-end route is the best delivery time because there are no additional delays through the transportation process. However, all the other cases deliver crude oil in the required time. Consequently, there is no delay at the end of transporting crude oil to the customer. The PTS is beneficial in that it saves transport costs.

**Figure 9:**
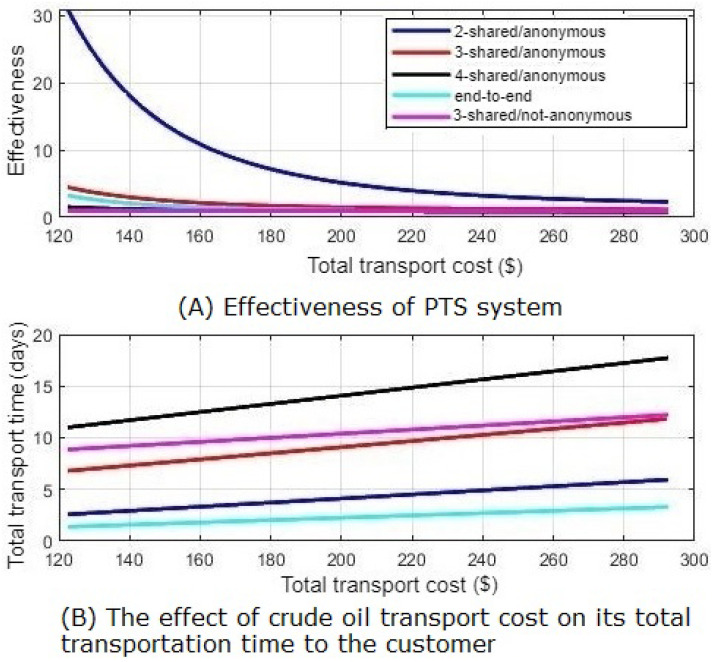
System effectiveness.

As a result, there is a trade-off between total transport cost and total transport time. As illustrated in Figure 9B, the selection that fits the minimum transport cost has the worst delivery time. However, the PTS system enables the exchange of petroleum products. Furthermore, it indicates that increasing product exchange processes through the open ocean enhances the PTS system’s effectiveness, which is its main objective. In addition, Figure 9A shows the system effectiveness $PTS_{eff}$. The effectiveness is calculated as below.

(11) $$PTS_{eff}=\frac{Cost_{Achieved}}{Cost_{Minimum}}$$

PTS’s effectiveness shows the achieved crude oil transport cost according to the system selection compared to the current transportation process cost. It illustrates how the PTS enhances the crude oil total transportation cost which is one of its vital challenges. As shown in Figure 9, the three shared non-anonymous customers pay the minimum cost with the effectiveness equal to 1, which means that its cost almost equals to the transport cost of the current process. Otherwise, the other different cases costs are greater with effectivenesses greater than 1. However, their effectivenesses are slightly greater than 1, except the two shared anonymous customers. Its cost are dramatically greater than the minimum cost, due to the added cost due to the anonymity mode.

**Figure 10:**
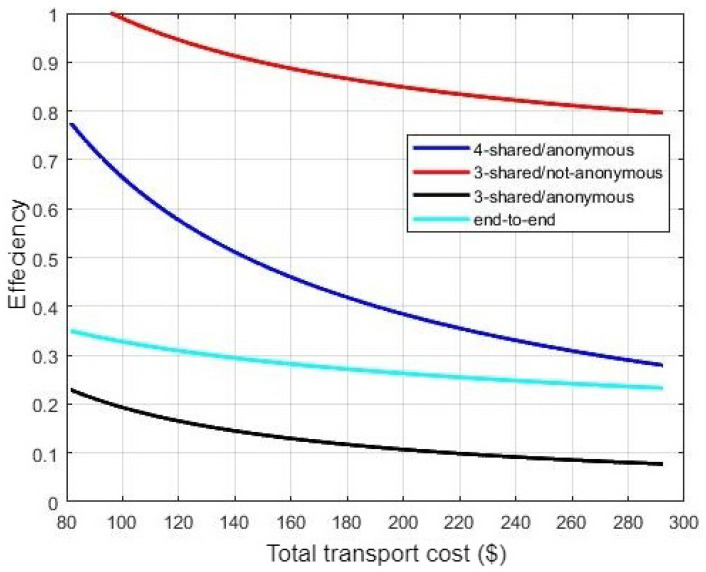
System Efficiency.

Figure 10 shows the system efficiency, indicating that PTS selects the optimal transport cost in addition to several vital options. Such options enable product exchange processes through the open ocean. As illustrated in Figure 10, PTS efficiency is better than the current case (end-to-end route) when increasing the number of shared customers. This is because it enables more product exchange in addition to minimizing the crude oil transport cost. In addition, as shown in Equation (10), PTS efficiency depends on the transport cost compared to the current crude oil transport cost. As illustrated in Figure 7, the case of sharing the transportation process with three non-anonymous customers presents the best transport cost, so PTS efficiency is the best in this case. The cost in the case of non-anonymous transportation process is better than the anonymous one as it does not add any additional cost for insurance. However, anonymous transportation enables product exchange processes through the open ocean due to the lack of identification restrictions.

Consequently, PTS improves the crude oil transportation process and enables more product exchange processes which increases the profit of crude oil companies and benefits the national income.

## 7. Conclusions and Future Work

This paper presented a novel Industry 4.0 PeTroShare (PTS), which integrates IoT networks, a ride-sharing system, and the blockchain concept, ensuring crude oil remains a public commodity. In addition to this, PTS created a new market for petroleum products by canceling the role of the broker in the petroleum industry. PTS used IoT networks for location tracking and quality control, while the integration of a ride-sharing in the PTS system enabled the exchange of multiple client petroleum products in the open ocean. In addition, blockchain preserved anonymous transactions within the crude oil transportation process. The searching algorithm found the optimal balance among all customers’ needs. It focused on determining an efficient and optimal tanker in addition to optimizing the total service cost. The evaluation results showed the effectiveness and efficiency of PTS. Furthermore, PTS can potentially enhance the transportation process efficiency. Our future work includes resolving security issues for a better transportation process.

## Figures and Tables

**Table 1:** Comparative analysis of the literature.

	The Related Work	PTS Model
[11]	The authors used profit decision models to compare transportation costs while using different types of ships. Unless the ice conditions allow safe navigation without using an icebreaker, the best solution is not to reduce the sailed distance but instead look for the fastest shipping lane.	In the presented model, PTS integrates IoT as a monitoring system assists in selecting the optimal tanker for the customer with efficient cost. Moreover, IoT sends alarm signals to the nearest convenient tankers to help handle the oil leakage problem.
[19]	The authors proposed a layered framework BCTLF that integrates IoT and blockchain in transportation and logistics to make it efficient and resilient against several security attacks.	PTS did not only use blockchain for security attacks. PTS also used it for making anonymous transportation processes. PTS succeeded in obtaining a new market for crude oil through the open ocean.
[20]	The authors provided the current status of IoT deployment in the oil and gas industry. The existing research projects were mainly run independently. They suffered from a lack of collaboration between different technologies.	In the presented PTS, the cooperative communication between IoT, ride sharing, and blockchain in the crude oil transportation process enabled many improvements such as improve quality of service, more accessible data collection, more automated industrial systems, and optimized cost expenditure.
[21]	The authors suggested a conceptual framework utilizing blockchain and smart contracts to monitor the overall oil supply chain. To store the smart contracts, they used two types of blockchain: permissioned and consortium. Delay in the update process can eventually cancel the smart contract.	PTS integrates ride-sharing technology in the transportation process to decrease the total cost. Additionally, PTS exploits IoT as a monitoring system to select the optimal tanker for the customer with efficient cost.
[22]	The authors proposed an economic traveling distance (ETD) approach to quantify each vehicle type’s best traveling distance range using its fuel consumption rate.	PTS presents the Industry 4.0 blockchain-managed, logistics-aware, privacy-friendly, cooperative, and efficient petroleum transportation platform.
[23]	The authors developed an operational strategy in which urban rail transit is used for freight transport. Freight can be transported by inserting dedicated freight trains or utilizing the extra space inside the passenger train carriages. Station platforms can load and unload both goods and passengers.	PTS uses a ride-sharing platform that enables an anonymous exchange of crude oil between oil producers and customers through the open ocean.
[13]	The authors studied the ride-share matching and routing problem in a nonprofit P2P ride-sharing system consisting of a matching agency, drivers, and riders.	PTS uses ride sharing to perform product exchange through the open ocean with more convenient routes.

**Table 2:** List of notations.

Notation	Definition
$S_{r}$	Set of customer requests
$S_{o}$	Set of crude oil rigs
$S_{t}$	Set of tankers
$Q_{a}$	Available Quantity
$Q_{r}$	Required Quantity
$L_{o}$	Location of crude oil rigs
$C_{f}$	Conflict factor
$T_{sub}$	Request submission time
$T_{travel}$	Time required to deliver crude oil
*T*	Needed by
$T_{wait}$	Waiting time
*F*	Regular Fare per mile
$D_{1}$	Travelled Distance by tanker
$D_{s}$	Travelled Distance by customer vessel
$N_{SC}$	Number of shared customers
α	Anonymity factor
$R_{c}$	Customer Request
*A*	Average matrix
*n*	number of crude oil rig
$C_{t}$	Crude oil tanker
$L_{C}$	Customer destination
$O_{r}$	Crude Oil rig
$\eta_{PTS}$	System Efficiency

**Table 3:** PTS simulation parameters.

Parameter	Value
Regular fare per mile	4.75
Tanker speed	18.64 mile/h
Vessel speed	24.86 mile/h
Crude oil flow rate	10.47 kg/s
Added cost	range of $500
